# Registration

**Published:** 1850-12

**Authors:** 


### REGISTRATION.

We are very glad to see that Dr. W. L. Sutton, of Georgetown, Ky., is energetically devoting himself to the procurement of the passage of a law for the registration of births, marriages, and deaths, within the Commonwealth of Kentucky. Of the importance of a well regulated and properly executed registration law, no man, with anything like a sound judgement, can entertain a reasonable doubt. We have a fear, however, that the law will not be either properly regulated or executed, and that the present race of physicians in Kentucky, will continue through life, to grope in the dark, or what is equivalent to it, stumble through the labyrinths of error on the subject of vital statistics. These are not reasons against the passage of a registration law but rather a plea for it. Let the work be commenced, and trust to the future, the improving influences of time, the beneficial lessons of experience, and the light that springs up along the paths of error sometimes, for the correction of what may be wrong in the law. We hope that every physician in Kentucky will bestir himself on the important matter of a registrat on law, and lend his influence to its passage. If they all act, as all should act on the subject, it can easily be attained.

There is not a human being in the commonwealth, no matter how obscure, no matter how remote from the active agencies of civilizattion, who is not interested in a complete registration law. It is a picture of progress; it teaches all the truths embodied in the phrase—VITAL STATISTICS, and who is not interested in that, and it enables generations to compare themselves with one another, on reliable data. These are the general results, but how much more important do they grow when we enter into details! We shall learn for the first time, what is the average length of life in Kentucky; we shall learn to distinguish between unhealthy and sanitary locations, and above all, we shall be enabled to ascertain the effect of the various pursuits of men and women, upon health. These elements of information will be worth to the State all they will cost.

Statistics of such value as these will possess, must be well paid for in order to insure fullness and accuracy; the registration officers must not only be well paid for their services, but in selecting them for the work, it must not be assumed that militia colonels are the only persons qualified to attend to this important business.

The great end and aim of Registration tables are thus set forth by Dr. Mauran, chairman of the Rhode Island Registration committee: "*Firstly*, to identify fully and conclusively every individual who is born, marries, or dies in the community,—for genealogical and municipal purposes. *Secondly*, to demonstrate results of age, profession, occupation; climate, season, and residence, upon the great subject of health. life and longevity—objects of the highest importance to every individual in society."

We entreat physicians throughout Kentucky, to awaken to this important matter. Let each one exert an influence upon his special representative in the legislature, and thus secure the great work in hand. B.

